# Design of 3D-printed hydroxyapatite intervertebral fusion cages in a patient specific framework

**DOI:** 10.1007/s11517-026-03546-8

**Published:** 2026-03-16

**Authors:** Anna De Cet, Martina Colombo, Luca D’Andrea, Ilaria Rota, Luigi La Barbera, Pasquale Vena, Dario Gastaldi

**Affiliations:** 1 Department of Chemistry, Materials and Chemical Engineering “Giulio Natta”, Laboratory of Biological Structure Mechanics (LaBS), Politecnico di Milano, Piazza Leonardo da Vinci 32, Milan, 20133 Italy; 2 Department of Biomechanics, Faculty of Medicine, Karl Landsteiner University, Dr. Karl-Dorrek-Straße 30, Krems, 3500 Austria

**Keywords:** Finite element analyses, Hydroxyapatite, Spinal fusion, Microstructure

## Abstract

**Abstract:**

Fusion surgery involves replacing degenerated intervertebral discs with artificial implants, usually composed of titanium alloys or PEEK, requiring bone grafting to assist tissue growth. This study aims to evaluate numerically 3D-printed porous ceramic implants as a bioactive alternative. Clinical computed tomographies of an adult patient were used to reconstruct the geometry and to assign mechanical properties to L1 and L2 vertebrae, utilizing a patient-specific anisotropic micromechanics-based model. The influence of different microstructural choices on a porous hydroxyapatite-based scaffold was analysed through finite element analysis, simulating standing and flexion. The analyzed scaffolds included: microstructured Face Centered Cubic (FCC) and Kelvin-based devices with uniform porosity (75%), and a Voronoi microstructured scaffold with uniform (75%) and graded porosity (60% external, 90% internal). Homogenized models were considered as a potential strategy to reduce computational costs. The FCC geometry and graded Voronoi proved more mechanically resistant to failure. Homogenized mechanical properties simplified the model, but didn’t accurately represent microstructural behaviour and local mechanical failure couldn’t be suitably identified. Patient-specific models allowed for a more accurate representation of mechanical stresses, leading to a reduced risk of structural failure. Hydroxyapatite proves to be a promising material for 3D-printed lumbar interbody fusion cages, able to provide primary stability.

**Graphical abstract:**

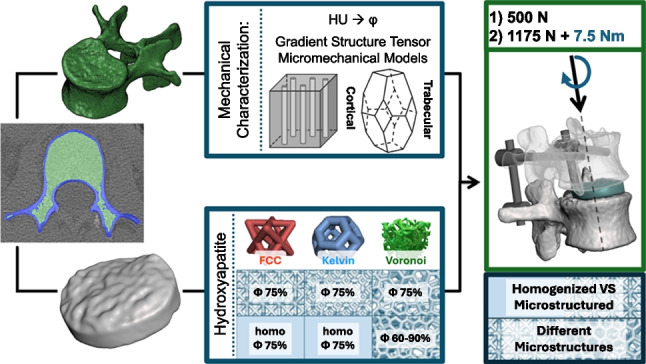

## Introduction

Either following a traumatic lesion or degenerative changes, the intervertebral disc may loose its ability to effectively redistribute loads between vertebral bodies, leading to pain, instability and potential damage to the spinal cord [1–4]. Intervertebral fusion surgeries aim to ensure that the primary cause for pain is removed, and to prevent further progression through effective immobilization [1, 2].

The clinical gold standard for fusion surgery involves auto-grafts, often using the iliac crest [5–8]. However, given the well-documented issues associated with auto-grafts [1, 7, 9, 10], intervertebral cages are now frequently utilized. These devices, produced in either titanium alloys or Polyetheretherketone (PEEK), aim to support and stabilize the spine as osteointegration takes place [1, 2]. To further aid bone growth, autologous or allogenic bone grafts are inserted in the cages [6, 11–13], therefore only partially compensating the need for self-transplants and, in cases involving allo-genic grafts, still potentially leading to infections and implant failure [9].

Titanium alloys are often utilized for bone substitution implants and devices, as it is durable and biocompatible. However, its elastic modulus is much higher than that of bone, leading to stress shielding phenomenons, implant loosening and failure [1, 14, 15]; furthermore, it is not bioactive and post-operative computed tomographies (CT) scans are affected by artifacts, which impede the evaluation of bone regrowth.

PEEK is characterized by high mechanical resistance and is overall mechanically similar to bone [16]; it is lightweight, biocompatible [17], and radiotransparent, allowing for follow-ups to be easily carried out [2, 18]. It is however unable to actively support osteointegration [1] unless proper coatings are implemented [19], and it is generally associated with a higher rate of device failure and subsidence compared to Titanium-based cages [20, 21].

Ceramics have been found to possess high potential for bone tissue regeneration [13, 22–27]. These materials are biocompatible [28] and osteoinductive, therefore they do not require the presence of bone grafts [28, 29], and are radio-opaque [30].

Syntetic Hydroxyapatite (HAp, $Ca_{10}(PO_4)_6(OH)_2$) has been frequently investigated due to its chemical similarity to mineral bone tissue components, allowing it to be resorbed as bone grows inside the implanted structure [23, 30, 31]. Clinical studies have reported on the utilization of HAp and HAp mixed with Beta-tricalcium Phosphates, both mixed with bone marrow: in both cases bone regeneration proved to be effective and comparable to traditional self-transplants [32, 33].

A downside of HAp is its intrinsic fragility [23]. This behaviour is worsened by the need to introduce pores in the structure, to ensure vascularization, cells’ seeding, migration, and bone ingrowth.

A balance between porosity and sufficient mechanical properties needs to be achieved, therefore requiring an accurate design that takes into consideration parameters such as device microstructures [34, 35] and material properties in relation to the production process [36].

Furthermore, it has been shown that tailored devices lead to safer solutions [29]: the patient-specific macroscopic shape of the device [37, 38], the loads the device will be subjected to [39] and how primary stability of the implant will be ensured should also be considered.

To design and test these devices, particularly in relation to their load-bearing capabilities, Finite Element (FE) models have been often utilized in literature [34, 35, 40], while, given all the degrees of freedom required by this design process, Additive Manufacturing appear to be the most convenient.

To develop FE models, certain assumptions can be implemented, such as simplified load cases, standardized geometries, and isotropic homogeneous bone mechanical properties. This latter simplification may play a significant role when the design process involves the evaluation of patient-specific bone-device interaction, as the biomechanical differences between cortical and trabecular bone and their anisotropic nature is well documented in literature.

To represent patient-specific bone anisotropy, $\mu$-CT based models are the most accurate [41–43], given the possibility to capture bone morphology to the trabecular level. However, the size of the scanning volume and the radiation dose involved prevent this method from being applicable in standard clinical practice.

Double detector CTs are utilized pre-operatively, but are often characterized by much lower and anisotropic resolutions. Studies have evaluated different methods for acquiring accurate and relevant information from low resolution CTs analysis [42, 44–48] and the implementation of micromechanics-based models [49–51].

The aim of this paper is to identify the most suitable microarchitecture of a porous ceramic scaffold for lumbar intervertebral fusion in a CT-based patient-specific model, through the implementation of homogenized or microstructured mechanical properties. Particular attention was reserved for the site-specific anisotropic biomechanical properties of bone tissue estimated from the patient’s CTs.

## Materials and methods

The general workflow of this paper was applied to one chosen dataset of clinical CTs belonging to an open source database [52–54].

First, anisotropic site-specific mechanical properties were estimated based on local Houndsfield Unit (HU) values and their spatial distribution. Then, a patient-specific intervertebral fusion porous cage, along with its fixation device was designed; the patient-specific nature of the model relates both to the geometrical and mechanical features of the bone tissue portion, which was tailored to the dataset, and to the device, which was designed in order to fill the space between two adjacent vertebras.

The macroscopic effect of introducing homogenized properties for the fusion cage, utilized to reduce computational costs, was compared with the results obtained when analyzing microstructured scaffolds with meshed struts. Then, four devices were computationally tested by implementing different chosen microstructures (FCC, Kelvin and two Voronoi-based lattice structures) and introducing a porosity gradient.

### Materials: Images dataset

The CT dataset utilized to generate the patient-specific computational model belongs to the collection of multidetector CTs made publicly available for the VerSe 2020: Large Scale Vertebrae Segmentation Challenge [52–55]. The specific dataset utilized (corresponding code 620) is not calibrated and belongs to the *Private Test subset*, which is noted to be composed of 45% female patients and characterized by a mean age of $57.9 \pm 17.6$; traumatic fractures and bony metastases were excluded from the original dataset selection. The specific patient dataset utilized was marked as belonging to a 19 years old male; indications regarding height and weight are not reported, both of which could offer additional relevant information in relation to patient-specificity. Though the entire spinal column was present in the original dataset, the volume was cropped to only the L1-L2 level. The initial anisotropic spatial resolution was comparable to standard clinical scan resolutions and corresponded to $0.326\times 0.326\times 0.6$ mm.

### Dataset preparation

The clinical resolution dataset was processed in the open-source software *3DSlicer* (https://www.slicer.org/) [56] . First, it was rescaled in order to transform the initial anisotropic spatial resolution ($0.326\times 0.326\times 0.6$ mm) through the application of a 3rd degree B-spline interpolation to obtain a cubic isotropic resolution of 0.326 mm^3^.

The semi-automatic segmentation process involved the following steps for each of the two vertebrae: (1) threshold-based definition of the volume of interest corresponding to the entire vertebral body, (2) threshold-based separation of cortical and trabecular bone, (3) manual edits to refine each segmented volumes. When separating the trabecular and cortical portion, particular attention was dedicated to ensure the continuity of the cortical shell, as certain portions of the cortical bone were characterized by a thickness comparable with the image resolution. These sections were therefore not characterized by high enough HU values to be separated from trabecular bone through simple thresholding.

Split cortical and trabecular segmentations were necessary to implement the voxel-by-voxel evaluation of HU values, which allows for the estimation of micromechanics-based mechanical properties that are then mapped onto a 3D mesh and implemented in the complete final model. The steps described in the following sections 2.3, 2.4, 2.5, 2.6 were carried out for each of the two selected vertebras.

### From HU to porosity

When developing a patient-specific computational model, the CTs utilized are calibrated, allowing for a correlation between HUs and material density, from which the mechanical properties of the tissue are estimated based on the chosen model [41, 57]. In order to estimate the tissue porosity in the volume occupied by each voxel from its HU value, a procedure inspired by the one utilized by Toniolo et al. in [48, 50] was leveraged. First, the probability distribution best fitting the histogram of the HU values characterizing each complete volume of interest was defined. Then, the HU value corresponding to the cumulative frequency of 0.98 [50] was selected as a threshold: values of HU higher than $HU_{threshold}$ would be considered corresponding to dense cortical bone, characterized by null porosity.

The porosity of each voxel was then estimated based on two separate linear correlations, one for trabecular bone Eq. 1 and one for cortical bone Eq. 2.

1 $$\begin{aligned} \Phi _{trab}(HU) = \Phi _{mode,trab} + \frac{(HU - HU_{mode,trab}) \cdot (\Phi _{max,trab} - \Phi _{mode,trab}) }{(HU_{min,trab} - HU_{mode,trab})} \end{aligned}$$

2 $$\begin{aligned} \Phi _{cort}(HU) = \frac{(HU_{threshold} - HU)}{(HU_{threshold} - HU_{min,cort})} \cdot \Phi _{max,cort} \end{aligned}$$

Where:

- *HU* is the value corresponding to each voxel being analyzed,
- $HU_{mode,trab}$ is the mode of all HU values inside the segmented trabecular bone tissue,
- $HU_{min,trab}$ is the lowest value of HU inside the segmented trabecular bone tissue,
- $\Phi _{max,trab}$ is the assumed maximum porosity of a trabecular bone voxel: 0.92,
- $\Phi _{mode,trab}$ is the assumed mode value of porosity of a trabecular bone voxel: 0.85,
- $HU_{threshold}$ is the minimum value of HU corresponding with dense cortical bone, characterized by null porosity,
- $HU_{min,cort}$ is the lowest value of HU inside the segmented cortical bone tissue,
- $\Phi _{max,cort}$ is the assumed maximum value of porosity characterizing a cortical bone voxel: 0.5.

It should be noted that $\Phi _{max,trab}$ and $\Phi _{mode,trab}$ have been defined as the values best matching literature based data following a trial and error procedure.

### Stiffness direction assignment

In order to implement orthotropic and transversely isotropic material properties, a Gradient Structure Tensor (GST) analysis was carried out to define the three mutually orthogonal vectors defining, for each voxel, the direction along which the stiffness should be highest and lowest.

Following the process described by Kersh et al. in [42], the eigenvalues $\lambda _1 \le \lambda _2 \le \lambda _3$ and eigenvectors $\underline{e_1}, \underline{e_2}, \underline{e_3}$ of the normalized structure tensor $\widehat{GST}$ was calculated in a $7\times 7\times 7$ voxels volume around the voxel to be characterized.

The voxel-by-voxel GST evaluation was carried out separately for cortical and trabecular bone. When the $7\times 7\times 7$ analysis volume was selected for a voxel belonging to the cortical segmentation, some voxels could belong to the trabecular segmentation volume, or to the soft tissues external to the vertebra itself. If the analysis volume contained more than 25% voxels that were not part of the current bone tissue portion being characterized, the voxel was flagged as “isotropic”and no stiffness directions were determined in order to limit the effect of the boundary on the estimated mechanical properties. The chosen value for the analysis volume corresponds to 1 mm radius of evaluation; it results large enough to have statistical reliability in defining the relevant directions, and small enough to limit boundary effects.

### Voxel-by-voxel micromechanical model

Split micromechanical models were implemented to estimate mechanical properties for cortical and trabecular bone.

#### Cortical bone tissue

The model chosen to represent cortical bone was acquired from a study by Hellmich at al. [51], where the tissue is modelled as a mineralized orthotropic matrix paired with cilindrical water-filled inclusions, which represent the Havers canals in cortical tissue [58].

As previously mentioned, some sections of the segmentations were characterized as isotropic due to insufficient voxels for a proper and reliable definition of stiffness directions. In such cases, the chosen formulation coincided with an isotropic matrix filled with spherical inclusions, as described by Eshelby [59]; the bulk elastic modulus chosen for the isotropic bone matrix was 20 GPa, to match the highest stiffness of cortical bone reported in literature [51, 60].

#### Trabecular bone tissue

The model chosen to characterize trabecular bone is the modified version of a Kelvin cell formulated by Sullivan et al. in [61, 62], leading to a transversely isotropic material formulation.

Sullivan et al. have previously defined a general tetrakaidecahedron model for open-celled foams in terms of Young’s moduli *E* (GPa), Poisson’s ratios $\nu$ [61] and shear moduli *G* (GPa) [62]. All the equations reported in the studies are written as a function of the mechanical properties of the solid material ($E_0$) and the geometrical parameters of the structure itself, such as:

1. macroscopic cell sizes, cell length *D* and height *H*;
2. edge cross-section geometrical parameters, edge cross-section area *A*, moment of inertia *I*, polar moment of inertia *J*;
3. the geometrical deformation parameter,inclination angle of the struts $\theta$;
4. cell edge lengths, *L* for the oblique struts and *b* for the horizontal struts.

Herein, a circular cross-section was assumed for the cell struts, therefore all edge cross-section geometrical parameters depend on the radius *r*. In addition, the height *H* and width *D* of the unit cell, as reported by Sullivan et al. [61] are related to *L*, *b* and $\theta$. A geometrical restriction that was previously adopted to treat non-isotropic foams [63–66] further correlates the cell edge lengths *L* and *b* with the inclination angle of the struts $\theta$, forcing the cell shape to depend only on this latter parameter.

By implementing these assumptions, the number of independent parameters is reduced to three, one of which is related to the solid material.

The cell aspect ratio *R* is defined as *H*/*D* and is equal to $tan(\theta )$ by implementing the geometrical assumptions mentioned earlier. *R* was assumed as the ratio between the minimum and the maximum eigenvalues ($\lambda _1$ and $\lambda _3$), as obtained from Section 2.4. Therefore, the parameter $\theta$ is directly dependent on the morphologic anisotropy parameters obtained from the gradient-based analysis. An estimation of morphological anisotropy may be expressed based on the eigenvalues themselves, through the following equation:

3 $$\begin{aligned} DA_{morphologic} = 1 - \frac{\lambda _{1}}{\lambda _{3}} \end{aligned}$$

The relative density of the cell, which can be obtained from the porosity through $1-\phi$, is defined in relation to the solid and total volume of the cell itself, and is dependent on $\theta$, the oblique strut length *L* and the strut cross-section radius *r*.

The input parameters for each equation are therefore:

1. the morphologic anisotropy, or the ratio between the minimum and maximum eigenvalues obtained as described in Section 2.4, which defines the parameter $\theta$;
2. the voxel porosity, which influences the relative density and defines the parameter $\frac{r^2}{L^2}$ through the morphologic anisotropy;
3. the composing material’s elastic modulus $E_0$.

### From voxels to tetrahedral mesh

Once all the voxels belonging to the segmented volume have been characterized, the defined mechanical properties are assigned to each tetrahedral element composing the volumetric mesh of each vertebra. The method utilized to assign the values is the Nearest Voxel Strategy (NVS), which is based on centroid distance between the voxel and the tetrahedral elements, which has been previously revealed to be a suitable approach [50].

### Scaffold design

The macroscopic geometry of the scaffold was obtained based on the segmented geometry of the adjacent vertebrae, resulting in a $27 \times 32$ mm elliptical surface and 9 mm thickness, to ensure a perfect fit between the interacting surfaces. Maximizing the surface of contact between bone and device aims to generate a smooth stress distribution and reduce peaks at contact areas [67, 68].

**Fig. 1:**
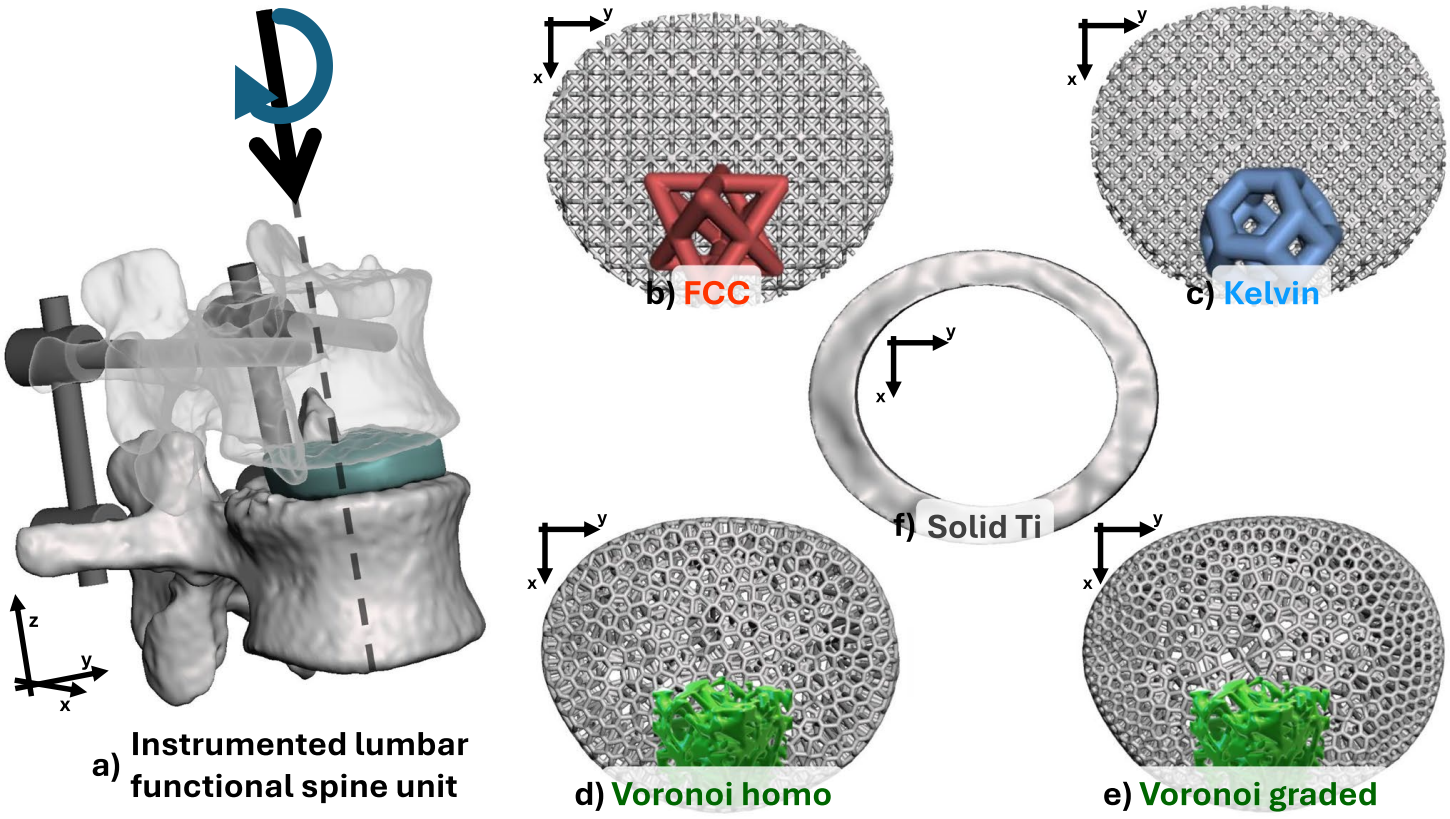
Summary of the complete model **(a)**, top-view of the different microstructured scaffolds implemented: (**b)** FCC-based with constant $75\%$ porosity, **(c)** Kelvin-based with constant $75\%$ porosity, **(d**) Voronoi-based with constant $75\%$ porosity, **(e**) Voronoi-based with graded porosity ($60\%$ external, $90\%$ internal), (**f**) ring-like Ti6Al4V-based for comparison

Three different microstructural geometries were chosen to generate a pattern to fill the device and introduce a porosity that will allow for tissue growth inside the device itself [69, 70]: face-centered cubic (FCC) cells, Kelvin cells and Voronoi-based microstructures. The introduction of these microstructures was obtained through the software *Ntopology* [nTop, Release 5.9.2, nTop Inc., https://ntop.com] and are reported in Fig. 1 b, c, d, e.

All three microstructures were implemented as meshed microstructures inside the volumes, in order to obtain a uniform porosity of 75%.

Additionally, a porosity gradient was implemented for the Voronoi-based microstructured device; a radial porosity, varying from 90% internally to 60% externally, would allow for the external areas to be able to better sustain physiological loads, while the internal core of the device would favor osteointegration through increased porosity. This was achieved by implementing a graded density of the Voronoi seeds inside the scaffold space.

Given the repetitive cell-based nature of the FCC and Kelvin geometries, the mechanical properties of a single cell were calculated and implemented in a homogenized version of the model through the *Homogenize Unit Cell* block present in the software *Ntopology*; this function calculates a unit cell’s effective mechanical properties, which can then be exported as a stiffness material matrix. These homogenized devices were implemented in this study in order to evaluate the efficacy of this modelling strategy in representing the microstructured device’s macroscopic and damage-related behaviour, while reducing the computational costs of the microstructured simulation.

A fully-solid Ti6Al4V cage, representing a standard interbody fusion device typically evaluated in literature, was also analysed as a comparison. Its design was obtained by boolean subtraction of the inner core of the entire scaffold, resulting in a ring-like structure with an average wall thickness of 3.8 mm, corresponding to $12\%$ of the device’s maximum diameter.

The average mesh size for all the microstructured devices was 0.15 mm, while the average mesh size for the homogenized devices was 0.4 mm. Additionally, the meshed microstructures were characterized by an average thickness of 0.44 mm for the Kelvin and FCC-based geometries and 0.5 mm for the Voronoi-based ones. The mechanical properties of the constituent materials and homogenized parameters are reported in Table 1.

**Table 1:** Mechanical properties assigned to the different devices [36]

Material	Elastic Modulus [GPa]	Maximum Tensile Stress [MPa]	Maximum Compressive Stress [MPa]
dense HAp	100	100	350
Kelvin-based HAp cell	4.88	1.89	6.62
FCC-based HAp cell	5.10	1.89	6.62

Seven different scaffolds were therefore analyzed: two homogenized with uniform porosity (FCC and Kelvin), three microstructured with uniform porosity (FCC, Kelvin, Voronoi), one microstructured device with a porosity gradient (Voronoi), and a Ti6Al4V-based device.

### Finite element simulation

In accordance with clinical practice, a Ti6Al4V stabilization system was designed and implemented in the model. Screws were simplified as cylinders with 5 mm diameter and 50 mm length, and connected in pairs through two 5 mm diameter and 50 mm length spinal rods. The final configuration of the model appears as reported in Fig. 1, comprising of: two vertebras (L1 and L2), four pedicular screws connected by cylindrical beams, and the scaffold being analyzed.

Both the screw-bone and device-bone interactions were simplified as tied constraints, assuming a stable interaction between all sections of the model. Additional simulations were run with the inclusion of a 0.3 friction coefficient [71] between the implanted device, bone tissue and the fixation system, to gain insight into its effect. More specifically, this friction was included for the Kelvin-based microstructure, both for the homogenized version of the device and for the microstructured one.

Two physiological loading conditions were considered to computationally test the device [72], as shown in Fig. 1:

- *Standing*, a vertical axial load of 500 N directed along the baricenters of the vertebrae [73],
- *Forward Flexion*, a vertical load of 1175 N combined with a bending moment of 7.5 Nmm directed transversally to the vertebrae [74, 75].

Both loads were applied to a reference point above the superior surface of the vertebrae, which was coupled with a selected area at the top of the L1 vertebra.

### Post-processing

Two main comparative analyses were carried out. First, the efficacy of implementing homogenized mechanical properties was analyzed: the macroscopic behaviour of the two devices was compared, in terms of stiffness and segment displacement, and the local damage distribution, based on strain values, was evaluated. Then, the different microstructures and porosity combinations were evaluated; in order to define the most suitable one for this specific fixation case, macroscopic stiffnesses, local damage amounts and distributions were observed.

The different parameters that were evaluated for these analyses are reported in the following paragraphs.

First, the volume of failed material inside the scaffold during *Forward Flexion*, computed as the percentage of volume whose maximum (and minimum) principal stress is higher than a specific threshold. For the microstructured scaffolds, the threshold coincided with the ultimate tension (and compression) stresses ($\sigma _0$) of dense HAp [76]. For the homogenized scaffolds it was the macroscopic values obtained through the Eq. 4 [77] .

4 $$\begin{aligned} \sigma = 1.21 \cdot (1-\phi )^3 \cdot \sigma _0 \end{aligned}$$

Here, the macroscopic stress $\sigma$ depends on the maximum material stress $\sigma _0$ and the scaffold porosity $\phi$. The specific values utilized are reported in Table 1. The higher the percentage, the higher the risk posed by the device to fail. The failed volume associated with compression and tension was computed both singularly, by considering the damage caused by compression stresses only and tension stresses only, and by looking at the overall damage, regardless of the type of stress acting on the volume itself.

The volume of damaged trabecular bone tissue during *Flexion* was also evaluated. It was computed as the percentage of volume whose minimum principal strain is lower than that of vertebral trabecular bone: $\epsilon _{trab} = 0.84 \pm 0.06\%$ [78], and quantified the negative effect of the device on the surrounding tissue.

During *Flexion*, the ratio between the material’s yield stress ($\sigma _Y = 795$ MPa [79]) and the maximum stress in the central portion of the two fixation beams was calculated. The strains in that same areas during * Flexion*, and the resulting forces acting on a planar cut of the cylindrical bars themselves during *Standing*, were also analyzed as indicative of the loads carried by the posterior fixation system, to compare with the values found in existing literature.

Finally, the macroscopic system stiffness during *Standing* was calculated from the force-displacement curve, and the degree of rotation during *Flexion* was extracted from the reference point the forces and moments were applied to. These parameters should highlight a good degree of stability of the fixed L1-L2 joint, associated with a small rotation angle, and a sufficient elasticity of the system, to still ensure mobility and therefore sufficient mechanical stimulation to promote osteointegration.

## Results

### Anisotropic inhomogeneous biomechanical bone tissue

**Fig. 2:**
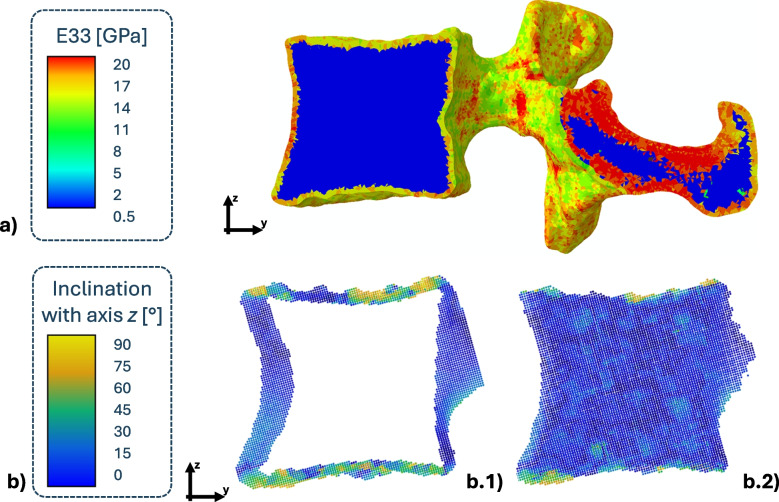
Colored maps of the defined material characterization: (**a**) maximum elastic modulus (E33) of cortical and trabecular bone tissue; **(b)** angle of inclination of the maximum principal elastic modulus direction, with respect to the vertical axis z, for b.1) cortical bone and b.2) trabecular bone

The values defined for the biomechanical properties of cortical tissue are equal to the following averages: $17 \pm 2$ GPa for the highest elastic modulus component, $9.5 \pm 2$ GPa for the median and $8.5 \pm 2$ GPa for the lowest. The direction coincident with its main elastic modulus was mostly aligned with the vertical axis of the vertebral body, as can be seen in Fig. 2, though a more horizontal inclination was present in the endplates.

Trabecular tissue was characterized as transversely isotropic and presented a higher elastic modulus component equal to $400 \pm 90$ MPa and a lower component equal to $160 \pm 40$ MPa. As defined previously in Section 2.5.2, the ratio between the higher and the lower elastic moduli is not constant, but depends on the morphological anisotropy obtained from the gradient-based analysis. As a consequence, the cell aspect ratio *R* is equal to $1.72 \pm 0.52$, which coincides with an anisotropy degree of $0.38 \pm 0.13$. The main elastic modulus direction recorded coincided with the vertical axis of the vertebral body.

### Microstructured HAp scaffolds

**Fig. 3:**
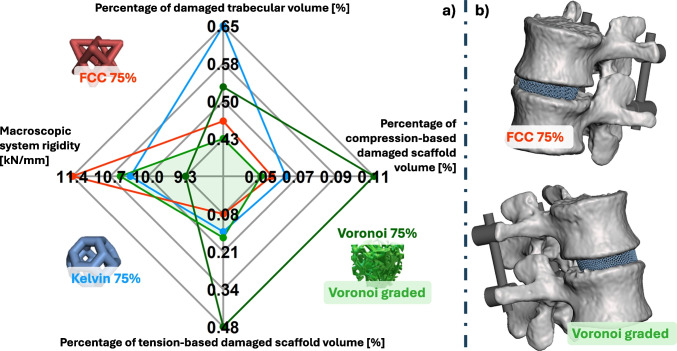
Comparison of different microstructures, based on chosen relevant parameters (**a**), and highlight of the two design chosen (**b**): the FCC-based uniform porosity and graded Voronoi-based scaffold

Figure 5 highlights, in its lower portion, the areas where damage is found based on the damage criterion previously established. As the results are reported for the *Flexion* loading condition, the damage is mainly located in the anterior portion of the scaffold and the vertebral body. Relevant chosen values in Fig. 3 graphically highlight the effects of the different microstructures. Furthermore, the exact values of each parameter related to the different devices are reported in Appendix A.

The percentage of failed volume in all microstructured devices averages to $0.28 \% \pm 0.21 \%$; this damage is mostly related to tension, as only $28 \% \pm 10 \%$ of the failed volume is associated with compression. It should be noted that the introduction of a graded porosity inside the Voronoi-based scaffolds did lead to a reduction in damage inside the device. More particularly, it was reduced by almost three times in terms of total damage. Furthermore, when the damage inside the inner and outer portion of the graded and uniform scaffolds was analysed, two things were noted: first, a slight reduction of damage inside the inner core ($0.20 - 0.19$), regardless of the significantly higher porosity characterizing the graded scaffold, and second a significant reduction of damage at the outer portion ($0.92 - 0.21$), as the graded porosity scaffold was characterized by a lower porosity than the uniform one.

**Fig. 4:**
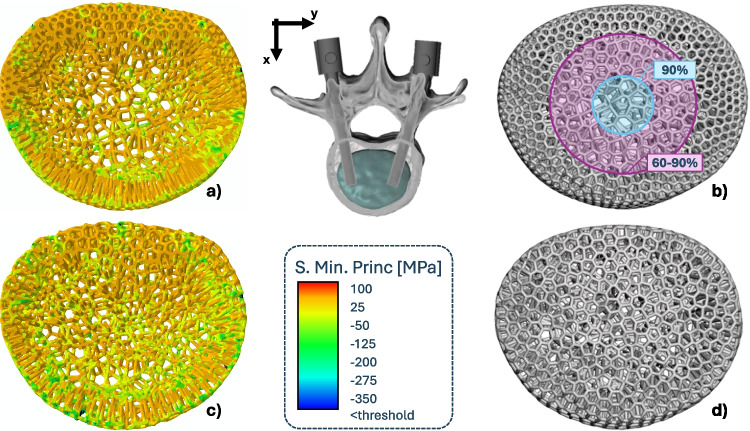
Comparison of uniform (**c**,**d**) and graded (**a**,**b**) Voronoi-based scaffold, reported both for a structural comparison (**b**,**d**) to highlight their morphological differences, and as colored maps of compressive stresses (**a**,**c**) for the *Forward Flexion* configuration

As the damage inside the scaffold was evaluated based on stress values, it was also observed that the volumetric averages of the stresses themselves were reduced in the graded device (see Fig. 4). It should be noted that both the graded and uniform porosity devices presented higher stresses at the outer portion of the device when compared to the inner core; however the graded device was able to perform better mechanically due to its reduced external porosity.

The volume of damaged trabecular bone varies in the different configurations but averages to $0.52 \% \pm 0.1 \%$.

The fixation system is characterized by maximum stresses equal to $64 \pm 7$ times the material’s yield value, and is therefore functioning in safe conditions. The minimum and maximum microstrains (strains multiplied by $10^6$) average to $-35.5$ and $-77$, and 54 and 105, for the left and right side. The load sustained by the fixation system during *Standing* averages to $4.1 \% \pm 0.2 \%$.

The macroscopic stiffness of the systems were equal to 10.35 kN/mm $\pm 0.85$ kN/mm; while the maximum angle of rotation reached was $5.5 \cdot 10^{-3}$ degrees $\pm 0.3 \cdot 10^{-3}$ degrees.

As mentioned previously, the effect of friction on the obtained results was evaluated.

The volume of damaged scaffold increased, reaching a value of 1.5, although the majority of the damage was still associated with tension stresses. Bone tissue damage also increased significantly, by a factor of almost 10. The fraction between the maximum stresses and the material’s yield value is significantly reduced during Flexion, while the percentage of load sustained by the fixation system itself during Standing is also lower ($<1\%$). The macroscopic stiffness of the system is increased.

### Homogenized HAp scaffolds

**Fig. 5:**
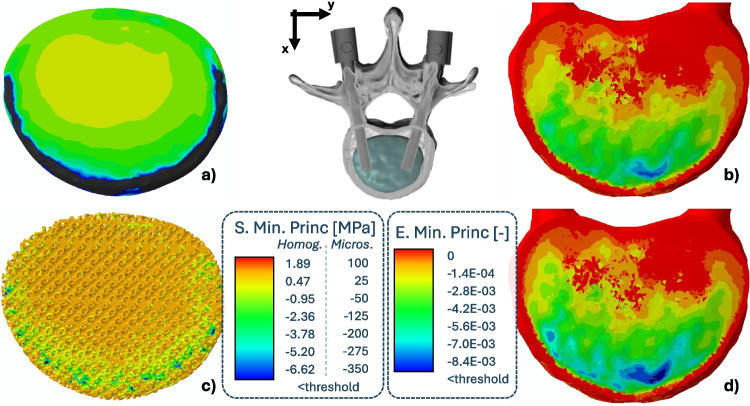
Top-view of the principal stresses and strains acting on the scaffold (on the left) and on bone tissue (on the right), for the Kelvin-based homogenized scaffold (top row) and the Kelvin-based microstructured scaffold (bottom row) during *Flexion*

In the upper portion of Fig. 5 the principal stresses and strains acting on the device are visualized, highlighting how their distribution varies when the microstructured devices are considered.

The percentage of volume of failed material inside the scaffold, during *Flexion*, corresponds to an average of $6.7\%$ and coincides with the dark area of Fig. 5 a. Furthermore, both homogenized scaffolds present much higher compression related damage ($90\%$ of the failed volume) when compared to tension related damage.

The percentage of trabecular volume characterized by principal strain values below the threshold previously defined was equal to $0.37\%$ for both homogenized scaffolds.

The fixation system is characterized by maximum stresses equal to 75 times the material’s yield value, therefore functioning in safe conditions. Microstrains in the central portion of the fixation beams reached a maximum compression strain of 34 and 66, and a maximum tensile strain of 50 and 90, for the left and right side respectively. The load carried by the fixation system during *Standing* equals to $3.5\%$ of the applied load.

The macroscopic stiffness of the construct is equal to 12 kN/mm, while the maximum rotation is negligible, corresponding to $4.8 \cdot 10^{-3}$ degrees for both homogenized devices.

The introduction of friction coefficients inside the model affected the results in the homogenized scaffolds also. Damage on the scaffold portion tripled, and the percentage of tension and compression damage is more evenly split. Damage on the trabecular portion is increased to $7\%$. As was the case for the homogenized scaffolds, the fixation system is characterized by higher stresses during Flexion, while carrying a lower percentage of load during Standing. The overall stiffness of the system is reduced by $30\%$.

### Solid Ti6Al4V-based device

No percentage of volume was reported to be damaged in the device, neither in compression nor in tension, while the percentage of volume of damaged trabecular bone corresponded to $0.31\%$.

The fixation system is characterized by maximum stresses equal to 135 times the material’s yield value, and is therefore functioning in safe conditions.The corresponding minimum microstrains were $-14$ and $-38$, while the corresponding maximum microstrains were 19.5 and 53. The load carried by the fixation system totaled to $3.2\%$.

The macroscopic stiffness of the system was equal to 14.59 kN/mm, and the maximum angle of rotation amounted to $3.9\cdot 10^{-3}$ degrees.

## Discussion

The aim of this paper has been to define the most suitable microarchitecture for a porous ceramic intervertebral fixation device, which was designed for vertebral L1-L2 fusion through a CT-based patient-specific model. Both homogenized or microstructured mechanical properties were implemented as a strategy to evaluate and rank their mechanical performance, and site-specific anisotropic biomechanical properties of bone tissue were estimated from the patient’s CTs.

The uniform FCC and graded Voronoi-based microstructures were able to achieve a good compromise between high resistance to damage and mechanical properties that should ensure a positive interaction with the surrounding tissue. Additionally, when comparing homogenized and microstructured representations of the devices, it was found that the homogenized scaffolds were sufficiently comparable with their microstructured counterparts in relation to their macroscopic behaviours, while the damage reported in the microstructured devices was significantly lower. Finally, the introduction of friction between bone tissue and implanted devices lead to a significant increase in damage on both elements. A single value has been chosen as representative for the evaluation this effect, and it could be considered as representative of a *worst case* condition due to its low value, therefore higher friction coefficients could be introduced.

### Anisotropic inhomogeneous biomechanical bone tissue

Vertebral bone tissue has been reportedly characterized as isotropic, with some studies assigning 15.7 GPa to cortical bone elastic modulus and 400 MPa to trabecular bone [73, 80]. The values defined in the current studies are slightly higher, though their average values are evaluated on the entire vertebral body, including the pedicles, which are characterized by the largest areas of high elastic moduli in Fig. 2, highlighted in red.

A study carried out on cylindrical samples of trabecular bone defined an average of $356 \pm 89$ MPa, which is in line with the values defined in our study [81].

Further evaluations could be carried out in order to define the level of accuracy of characterization achieved with this algorithm; direct comparison of $\mu$-CT scans and standard clinical resolution scans of the same sample, could highlight the differences between the stiffness directions defined and the mechanical properties being estimated.

### Standard solid Ti6Al4V-based device

Given the novelty of the algorithm utilized to characterize bone tissue properties and the scaffolds themselves, and the exclusively computational nature of the study, a Ti6Al4V-based device was implemented in the study, in order to define parameters to be compared with the existing literature.

The percentage of load carried by the posterior fixation system remains below $5\%$ in all devices during *Standing*. This is lower than what Khodaee et al. reported in [82], where the load sustained by an implanted device during pure compression was equal to around $10\%$ for a bilateral PLIF cage and $15\%$ for a TLIF cage. These discrepancies can be linked to multiple factors.

First, the absence of spinal ligaments in the current study’s model could be considered as a *worst case* condition, where the load supported by the soft tissues (i.e. ligaments and facets), estimated around 40 to $50\%$ respectively for TLIF and bilateral PLIFs cages [82], in the current study is sustained by the designed device. The conditions simulated herein are worse than having considered all anatomical structures.

Furthermore, the footprint of the device is larger and wider than both a standard Posterior Interbody Lumbar Fusion (PLIF) or Transforaminal Lumbar Interbody Fusion (TLIF) cage, since it has been maximised to ensure a more even load distribution and reduce fracture risks. Previous literature findings have highlighted that both increases in stiffness and size of the implanted cages significantly reduce the load carried by the posterior fixation system [83]. Furthermore, the stiffness of the implanted device heavily influences the loads carried by the posterior fixation [72]. Additionally, differences in spinal rod and pedicle screws designs, as well as differences in the considered spine segment, may explain some difference.

A computational study by Talukdar et al. [84] compared average Von Mises stresses in Ti6Al4V-based cages for the L4-L5 segment, evaluating how the introduction of uniform and graded microstructures would affect the mechanical performance of the device and the biomechanical stimuli assisting osteointegration. The device analyzed was however smaller, therefore the average Von Mises stresses reported inside the cages were higher than those found in the current study.

###  Homogenized scaffolds as a modelling strategy

Relevant differences were identified between the homogenized scaffolds and their microstructured counterparts, particularly in relation to damage, leading to the hypothesis that, in the observed configurations, homogenization is not sufficient to appropriately represent the mechanical behaviour of the device at a mesoscopic level.

It should be noted that the reported percentage of volume of failed scaffold was significantly higher in the homogenized versions compared to the microstructured ones, ranging from 4 to 100 times higher for tension-based and compression-based damage respectively. This behavior was also present when a friction coefficient was introduced. It must be emphasized that a reliable estimate of the macroscopic strength for a ceramic microstructure cannot be obtained for the intrinsic brittle nature of the material and for the complex interplay between loading direction and orientation of the microstructures [76]. Therefore, the analyses carried out on the microstructured model should be considered more accurate. The damage reported on the side of trabecular bone tissue was instead higher for the microstructured scaffolds when compared with their homogenized counterparts, though to a lesser degree (1.5 times in average). This is likely due to the struts that are locally stiffer than the softer homogenized material, thus, leading to strain intensification effects on the surrounding bone. A summarized graphical view of this comparison can be found in Fig. 5. In both cases, the damage was concentrated on the anterior portion of both the device and bone, which is consistent with the flexural loading.

A macroscopic evaluation of the analyzed devices lead instead to sufficiently similar results: both the macroscopic stiffness and the maximum rotation were comparable, and the percentage of load carried by the fixation system during *Standing* was only slightly higher (1.2 times) in the microstructured scaffolds.

Therefore, the choice between homogenized and microstructured devices should be guided by the design objective. If only the macroscopic elastic behaviour of the device is the main focus, then the homogenized representation is sufficient to capture the mechanical properties and is less computationally expensive. If the objective is to evaluate the effect on damage behaviour of design choices, as is the case for the current study, then the implementation of microstructured devices will ensure an improved representation of the device at the microscale. For HAp-based devices, in particular, an analysis of the high-risk areas of a microstructured device is needed to ensure the safety of the device and prevent catastrophic failures by specifically targeting high-risk areas [85, 86]. It should also be noted that the homogenization process did not take into account the incomplete nature of the cells present at the edges of the device, which is caused by the irregular shape of the device itself, and therefore some corrective factors could be implemented to improve on the mechanical representation [87].

### Microstructural comparison

A comparison of the different microstructural designs has been carried out, in order to define the effect of design parameters on the biomechanical performance of these devices. Though the values obtained are often comparable, some relevant chosen parameters highlighted more desirable microstructures. These are reported in Fig. 3 in the form of a radar chart, where the optimal condition is identified by the design that minimizes two out of the four parameters chosen.

Though the damage-related values of the different microstructures are all small, HAp-based devices are prone to catastrophic fractures before new bone is formed, providing additional strength and toughness. A single strut failing, then, leads to fast fracture propagation [88]. Therefore, smaller percentages of failed volume can still be considered as indicators when carrying out comparative analyses. In this regard, the FCC-based and graded Voronoi microstructures have displayed the lowest percentage of failed volume. The introduction of a graded porosity, in particular, has lead to a significant improvement with respect to the Voronoi-based device with uniform porosity, which manifested as a significant reduction of damage inside the entire device volume. This confirmed the initial assumption that the lower porosity in the external portion of the device would perform a “shielding” action by succesfully bearing the load without being mechanically compromised, due to the reduced damage recorded. Further improvements may be achieved by implementing a preferential direction to the distribution of microstruts inside the Voronoi geometries, mimicking the complex load-dependent behaviour of the native trabecular bone [89].

As for the comparison between homogenized and microstructured devices, the parameters related to macroscopic behavior were not significantly different between the four microstructured scaffolds. However, particular attention was given to the macroscopic stiffness of the system: though the aim of an interbody fusion surgery is to stabilize the adjacent vertebral segments, a certain degree of movement is required to stimulate osteointegration, and it has been reported [73] that cages that are less stiff are able to better stimulate the cellular substrate from which bone growth originates. Furthermore, clinical studies have proven that between softer PEEK-based cages and stiffer metal-based ones, PEEK cages tend to have higher fusion rates [90, 91], though this could be further improved through the implementation of titanium coatings. The voronoi-based microstructure being characterized by the lowest stiffness when compared with other microstructures is in line with previous literature [35].

It is important to note that in the literature studies above mentioned the implants were not accompanied by posterior fixation, and the materials composing the interbody cages were not fragile or did not display fragile-like behaviours, therefore a high degree of deformation could be achieved without increasing the risk of device failure. The devices evaluated in the current study all need posterior fixation to prevent excessive loading at initial stages, which would lead to breakage, and cannot display high levels of macroscopic deformation. Still, it is necessary to ensure sufficient mechanical stimulus to the surrounding native bone to allow for bone ingrowth, despite the increased stiffness of the entire system generated by the presence of the fixation system.

Though the FCC-based microstructured scaffold displayed overall the lowest damage-related parameters, its significantly higher stiffness may negatively affect bone growth.

Comparing the two Voronoi-based geometries confirms the hypothesis that lead to the introduction of the graded porosity: the lower porosity on the external portion of the scaffold assist in shielding the inner core from damage, leading to a reduction in the percentage of failed volume, while increasing the macroscopic stiffness.

The implementation of anisotropic tissue inside the model aims to achieve an increased complexity of tissue representation, as this study focuses on bone-device interaction. This may lead to an improved evaluation of how the loads are redistributed inside the system when compared with homogeneous isotropic material characterization.

One known limitation of this study relates to the bonded boundaries implemented for all interactions inside the model or the aim of comparing mechanical performance. This is an assumption that has been previously chosen in literature [92–94], but it does limit the evaluation of mechanical performance of the implanted device as the following phenomenons were neglected: the presence of micromotion in screw-bone interfaces and potential loosening, and micromotions between scaffold and bone. Furthermore, it could lead to overestimation of tensile stresses during flexion, particularly in the posterior portion of the intervertebral device. The additional analyses carried out highlighted how the loosening of this tied interactions through the introduction of a friction coefficient lead to an increase of damage inside both bone tissue and implanted device, and of the stresses and strains acting on the fixation system. Further evaluations regarding friction coefficients specific to the implanted devices should be carried out, as the printing strategies and pre-implant steps required may affect the surface finish of the implant itself.

It should be noted that the aim of this work was focused on the computational methodology, therefore the patient themselves was not assumed to be degenerated, based on the available data, and the main objective of the intervertebral fusion device was to substitute the intervertebral disk. The influence of surgical realignment of the spine was therefore neglected, and no vertebral realignment was carried out. The design process of an implantable cage should however take into account the angles and heights required by a surgical expert in order to ensure a correct spinal alignment.

Future studies may improve on the current results by evaluating more loading conditions, particularly torsion, and including soft tissue representation. As the aim of introducing ceramic-based scaffolds is to assist osteointegration, the effect of different microstructures on this process may also be evaluated by implementing numerical models of bone remodeling [95, 96]. Furthermore, this modeling strategy may be applied in the future at any lumbar level where a lumbar fusion surgery may be involved, as this strategy has proven its feasibility and its ability to offer insights into the effect of design choices on implantable devices. Additionally, this methodology should be applicable to thoraco-lumbar and lumbar-sacral fusion surgeries. This should be done while taking into consideration the necessary adjustments related to the physiological geometries of these sections, for example the increases intra-vertebral angle characterizing lower lumbar vertebrae, and if the cases analyzed are pathologic in nature, surgical planning insights should guide the macroscopic design of the implant.

## Conclusion

This paper has evaluated the effect of different modeling and morphological design choices on the mechanical behaviour of 3D-printed HAp-based scaffolds for L1-L2 intervertebral fusion, leveraging a patient-specific framework.

A homogenized representation of the device’s properties has proven to be a sufficiently accurate representation if the aim is to evaluate the macroscopic behaviour of the device itself, however a microstructured representation is required for a more accurate evaluation of the areas where damage is most likely to occur. The FCC and Voronoi-based microstructures have proven to achieve the best compromise out of the option evaluated, as they were characterized by the lowest damage levels while ensuring good mechanical stimuli to support bone regeneration.

This study therefore supports the applicability of HAp for load bearing applications, when the design of the device is carried out both macro- and microscopically, taking into account different morphometric parameters and ensuring that a compromise between different requirements is met.

## Data Availability

All data supporting the findings of this study will be made available on request.

## Appendix A: Microstructured data table

**Table 2:** Summary of the results obtained for all microstructured devices

	Kelvin 75%	FCC 75%	Voronoi 75%	Graded Voronoi (60-90)
Percentage of damaged scaffold [%]	0.20	0.13	0.59	0.21
Percentage of compression-based damage [%]	30.22	40.72	18.88	22.18
Percentage of damaged trabecular bone [%]	0.65	0.46	0.53	0.43
Percentage of damaged trabecular bone [%]	0.65	0.46	0.53	0.43
Safety factor (Left $\mid$ Right) [-]	123 $\mid$ 57	139 $\mid$ 73	118 $\mid$ 59	126 $\mid$ 68
Principal minimum microstrain (Left $\mid$ Right)	−36 $\mid$ − 87	−33 $\mid$ −67	− 37 $\mid$ −82	−36 $\mid$ −71
Principal maximum microstrain (Left $\mid$ Right)	55 $\mid$ 117	49 $\mid$ 92	57 $\mid$ 114	54 $\mid$ 99
Load sustained by fixation [%]	4.2	3.9	4.0	4.3
Macroscopic rigidity [GPa]	10.29	11.35	9.28	10.48
Maximum rotation [$10^{-3}$ $^{\circ }$]	5.5	5.1	5.8	5.4
